# Assessment of knowledge, attitude and practice about malaria and ITNs utilization among pregnant women in Shashogo District, Southern Ethiopia

**DOI:** 10.1186/s12936-015-0755-7

**Published:** 2015-06-04

**Authors:** Terefe G. Fuge, Samuel Y. Ayanto, Fiseha L. Gurmamo

**Affiliations:** Department of Medical Laboratory Sciences, Hossana College of Health Sciences, P.O. BOX 159, Hossana, Ethiopia; Department of Public Health, Hossana College of Health Sciences, Hossana, Ethiopia

**Keywords:** Malaria, ITNs, Pregnant women

## Abstract

**Background:**

Malaria causes variety of adverse consequences in pregnant women due to invasion of the placenta by Plasmodium. It increases the risk of adverse pregnancy outcome for the mother, the foetus and the new-born. Therefore, knowledge, attitudes and practices of this vulnerable group about malaria and the effective use of insecticide-treated nets (ITNs) contribute to sustainable control of the disease and its effects.

**Methods:**

A community based cross-sectional study was carried out in May, 2014. A validated structured questionnaire was used for data collection. The data was analysed using logistic regression by means of STATA version 11 data analysis software.

**Results:**

A total of 398 pregnant women participated in the study and their overall knowledge and attitude towards malaria and ITNs was fairly good; 74.3 % of the mothers had good knowledge and 51.1 % of them possessed positive attitude. Nevertheless, only 15.6 % of the mothers associated mosquitoes with malaria and majority of them (65.6 %) responded that it is transmitted due to poor personal hygiene and environmental sanitation. Younger age, receiving information and information obtained from health extension workers and media were found to be important predictors of pregnant women’s attitude (*P* < 0.05). The ITNs utilization was poor. Only 15.8 % of 398 mothers owned at least one ITN. This was due to its unavailability in markets and unsustainable distribution. More than half of the mothers who owned the ITNs did not have a number proportional to their family size, and 52 % of the mothers had not slept under bed net the previous night. This was due to its being dirty, old, had holes and in some cases lack of awareness on how to install it and its importance to prevent malaria. Higher education was identified as the determining factor for ITNs utilization (*P* < 0.05).

**Conclusion:**

Even though the pregnant mothers’ knowledge and attitude about malaria and ITNs was fairly good, its ownership and utilization was noticeably very low. Therefore, consistent and timely distribution by the government and other funding agencies is promptly needed. In addition, appropriate health education should be given on the link between malaria and mosquito, regular and correct use of ITNs with special focus to uneducated and elderly mothers.

## Background

Malaria poses an enormous burden to the world’s population, with 216 million cases and 655,000 deaths attributable to this mosquito-transmitted parasite in 2010 alone. The burden is largely borne by Africa where 91 % of deaths occurred, with pregnant women, their unborn babies and children under five years of age most at risk of infection and adverse outcomes [1]. These groups are at high risk due to weakened and immature immunity respectively [2]. Each year, there are an estimated 25 million pregnancies in sub-Saharan Africa at risk of malaria, the consequences of which can be serious for both mother and foetus in terms of morbidity and mortality [1–3].

Malaria during pregnancy is associated with adverse health outcomes such as maternal anemia, Intrauterine Growth Retardation (IUGR) and the delivery of low birth weight infants. Low birth weight (<2500 g) is considered to be the leading cause of death among infants in sub-Saharan Africa [4].

In Ethiopia, more than three-quarter of the landmass (altitude <2000 m) of the country is malarious, and about 68 % (>50 million people) of the total population is residing in areas at risk of malaria infections, pregnant women and under-five children being the most vulnerable groups. The Plasmodium species which have epidemiological importance in Ethiopia are *Plasmodium falciparum* and *Plasmodium vivax*; *Anopheles arabiensis* is the major malaria vector and it breeds in small sun exposed pools mainly produced during the rains. Malaria transmission in Ethiopia is unstable and characterized by frequent and often large scale epidemics. In 2010, the disease accounted for 98/100,000 admissions and 4/100,000 deaths [5–9].

The World Health Organization (WHO) recommends the use of insecticide-treated nets (ITNs) as a measure to reduce the mentioned adverse effects during pregnancy. Similarly, one of the goals of the National Malaria Strategy in Ethiopia is to ensure that vulnerable individuals such as pregnant women benefit from preventative measures, such as ITNs. Even though, the Abuja declaration targets agreed upon by African heads of state in 2000 aims to provide at least 80% of pregnant women with ITNs by the year 2005, only 63% of pregnant women presently make use of an ITN in Ethiopia which hampered the effectiveness of ITNs. This is mainly due to issues related to replacement of nets, seasonality of malaria, and poor knowledge with regard to the link between mosquitoes and malaria as well as proper utilization of ITNs [9–11]. Therefore, assessment of knowledge, attitudes and practices about malaria and the effective use of ITNs in this vulnerable group contribute immensely to sustainable control of the disease.

## Methods

### Study area

**Fig. 1:**
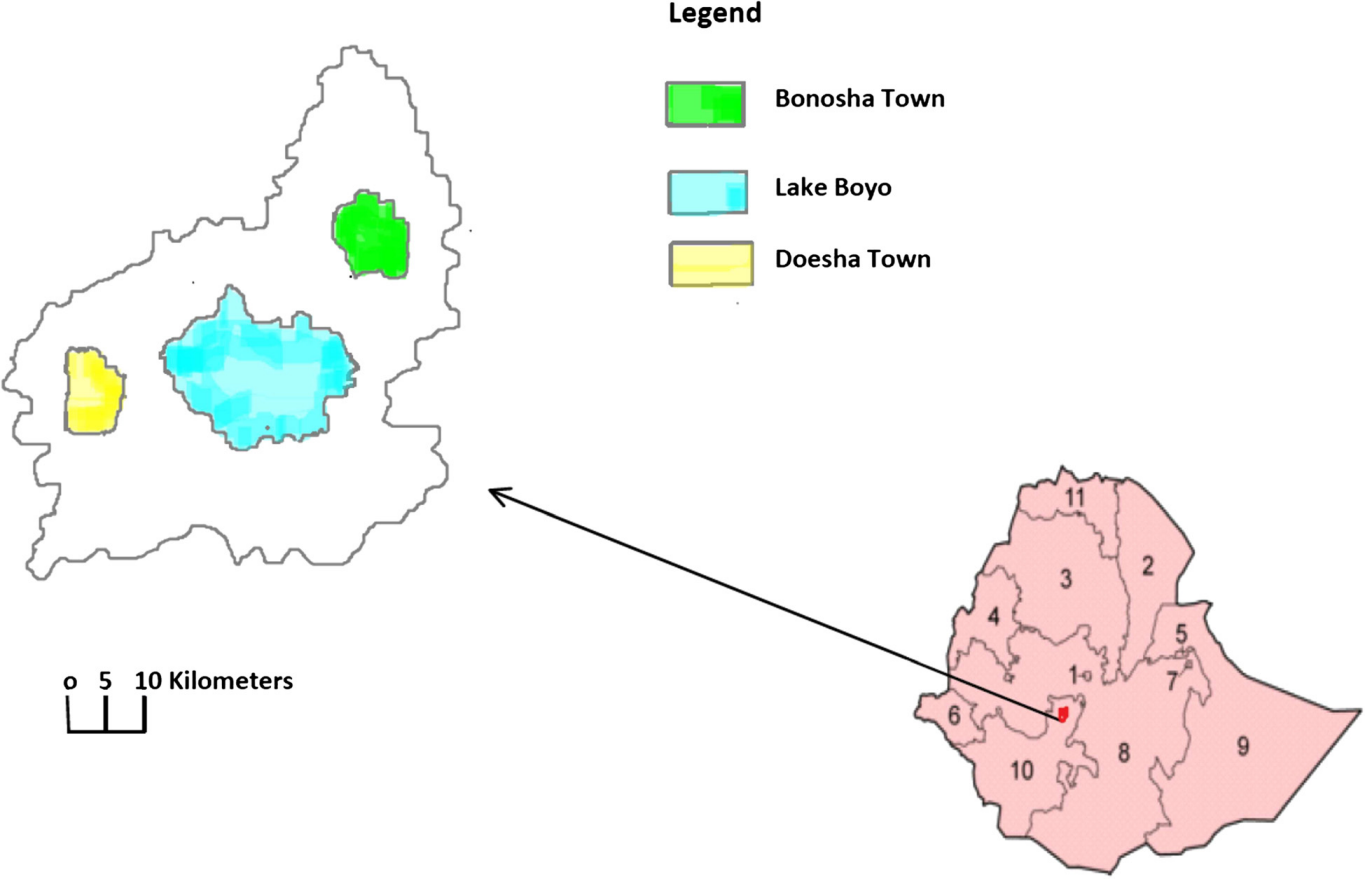
Map of the study area

The study was conducted in Hadiya Zone, Shashogo District, which is 224 km far from the capital of Addis, 117 km from Hawassa capital of Southern Nations, Nationalities and Peoples Region (SNNPR), 52 km from zonal capital Hossana at an elevation ranging from 1800 to 2000 m above sea level (Fig 1). The Woreda contains 36 *kebeles* (smallest administrative unit in Ethiopia) (34 rural and two urban) within an area of 32,310 km^2^ and it has a total population of 12, 7281, of which 4200 are pregnant women. Shashogo Woreda has predominantly dry kola (hot low land) agro-ecology. The rainfall has a bimodal nature in which the months from March to May and June to September are marked by relatively higher rainfall records; while months from November to February are dry. The long rainy season in the area is between June and September, during which crop cultivation takes place in the area. The total annual rainfall reaches 1005.1 mm. The mean maximum temperature is 21.6 °C, occurred during February; while the mean minimum temperature is 18.5 °C occurred during July. Water bodies such as streams and rivers commonly exist in the area. There is also a lake which surrounds two of the kebeles. The Woreda has five health centres and thirty-six health posts each with two health extension workers. Its health service coverage reached 98 % in 2013. The main malaria prevention and control strategies being undertaken in the Woreda include ITNs, IRS, use of larvicidal chemical (Abate), environmental modification and case management through early detection and treatment.

### Study design

A community based cross sectional study was carried out in May 2014.

### Sample size estimation and sampling technique

By using a formula for the estimation of a proportion: *n* = Z^2^. P(1-P)/e^2^ [12] and *P* = 0.5 (because of the approximation of pregnant women properly utilizing ITNs in the area is unknown), 95 % CI (1.96), 5 % margin of error (e) and contingency for non response rate of 10 %, a maximum of 422 study subjects were required. to obtain this, 422 households were randomly selected after obtaining a list and address (house number) of every pregnant women from health posts in each kebele. the number of households to be included in the study from each kebele was determined proportionally based on the population of pregnant women.

### Data collection instrument and method

A close-ended, pre-tested and structured questionnaire [13] was administered by interview method to all pregnant women visited at home. Early in the morning, each pregnant woman was visited to observe the actual practice regarding the use of ITNs during the previous night, and each selected household was revisited on the same day to answer the remaining questions. The questionnaire comprised four sections: Socio-demographic data, knowledge and attitude about transmission of malaria and its preventive measures, and ownership and use of insecticide-treated bed nets. The data was collected by four trained professionals having diploma in health related fields.

### Determination of knowledge, attitude and practice regarding malaria and ITNs

#### Knowledge

Common principles used to measure knowledge about malaria include questions about transmission and interventions [14]. This study used similar principles to generate eight multiple-choice questions, each of which was scored one point for a correct response and zero for the rest. An overall knowledge score was calculated by adding up the scores for each respondent across all nine questions. Those whose score are equal to mean score (5.9) or above were taken as having good knowledge while those with score of less than the mean were considered as having poor knowledge about malaria.

#### Attitude

By scoring five points for the right answer and one point for the wrong answer following the Likert’s scale, an overall attitude score was determined for each respondent by adding up the scores across the seven attitude questions. Respondents with score of greater than or equal to the mean score (28.5) were considered as having positive attitude whereas those with score of less than the mean score were taken as having negative attitude towards malaria and ITNs.

#### Practice

The seven questions that indicate malaria practices were scored for each respondent. If respondents indicated that she always performed a good practice i.e., sleeping under a mosquito net, she was given a score of two points. If she indicated that she sometimes performed a good practice, she was given a score of one point. If on the other hand, she indicated she never performed a good practice, she was given a score of zero. An overall practices score was determined for each respondent by adding up the scores across the seven ITNs practices questions. Respondents with score of greater than or equal to the mean score (6.1) were considered as having good practice while those with score of less than the mean score were taken as having poor practice in relation to ITNs.

### Ethical considerations

Consent was sought from the Woreda Health Office and informed consent was obtained from the study subjects. The study also obtained ethical clearance from the Research Committee of Hossana College of Health Sciences. Pregnant women who were looking ill were referred to the health centers for further diagnosis and treatment. Malpractices related to ITNs and non-utilization of ITNs was communicated through health education given after interview.

### Data analysis

The data obtained from the study was computerized using Epidata version 3.1 data entry format and exported to statistical software, STATA version 11 for analysis. Means and standard deviations was calculated for continuous variables while crude and adjusted Odds ratio (OR) was calculated to check statistical association between the dependent and independent variables using the binary logistic regression and multivariable logistic regression models. All variables of the study were initially tested for association with poor knowledge, attitude and practice regarding malaria and ITNs by using the binary logistic regression model. Those which show statistical association (*P* < 0.05) were put in the multivariable analysis model to check if the association existed after controlling against all the rest of the variables. All statistical tests and generalizations were done by assuming 95 % confidence interval and 5 % level of significance.

## Results

### Socio-demographic characteristics

Out of 422 women estimated to be included in the study 398 of them provided complete information while the remaining 24 were not found in the house during the date of interview or unable to complete the questionnaire. More than half, 215 (54 %) of the mothers were within the age range of 26–35 and those in the age range of 36–45 were rare i.e., 63 (15.8 %). The remaining nearly one third (30.2 %) was accounted by women of the age group 15–25.

The vast majority of the women were married 390 (98 %) with very few single, widowed and separated cases. A little more than three fourth, 305 (76.7 %) of the respondents had four or more family members including the pregnant woman. Almost half, 192 (48.2 %) of the women attended primary school but 168 (42.2 %) of them were unable to read and write.

Being a housewife was the main occupation for the mothers (93 %) with very few cases of civil servants and students. Protestant religion followers were high in number, 212 (53.3 %) followed by Muslims, 179 (45 %). Most of the pregnant women shared their sleeping furniture with other family members (95 %) and bed was their main sleeping furniture (65.6 %). 384 (96.5 %) of the respondents reported as they received information related to malaria and ITNs and their main source of information was health institutions (87.8 %) particularly health extension workers (Table 1).

**Table 1:** Univariate analysis of association between knowledge about malaria and ITNs and socio-demographic characteristics among pregnant women, Shashogo Woreda, Southern Ethiopia, 2014

Variables	Label	Frequency (%)	Good	Poor	Crude	*P*-value
			n (%)	n (%)	OR (95 % CI)	
Age	15–25^a^	120 (30.2)	86 (71.7)	34 (28.3)	1.00 (0.67–1.49)	0.85
	26–35	215 (54)	165 (76.7)	50 (23.3)	1.30 (0.95–1.79)	
	36–45	63 (15.8)	44 (71)	18 (29)	0.96 (0.56–1.67)	
Marital status	Single^a^	4 (1)	2 (50)	2 (50)	1.00 (0.14–7.10)	0.25
	Married	390 (98)	291 (74.8)	98 (25.2)	2.97 (2.36–3.73)	
	Widowed	3 (0.75)	1 (33.3)	2 (66.7)	0.50 (0.05–5.51)	
	Separated	1 (0.25)	1 (100)	0 (0)	--	
Education status	Illiterate^a^	168 (42.2)	119 (71.3)	48 (28.7)	1.00 (0.71–1.40)	0.32
	Read and write	9 (2.3)	6 (66.7)	3 (33.3)	0.81 (0.20–2.86)	
	Primary (1–8)	192 (48.2)	150 (78.1)	42 (21.9)	1.44 (1.02–2.03)	
	Secondary and above	29 (7.3)	20 (69)	9 (31)	0.90 (0.41–1.97)	
Occupation	Student^a^	9 (2.3)	6 (66.7)	3 (33.3)	1.00 (0.25–3.55)	0.17
	Housewife	370 (93)	272 (73.7)	97 (26.3)	5 (0.64–39.06)	
	Farmer	1 (0.2)	1 (100)	0 (0)	----	
	Self employed	7 (1.7)	6 (85.7)	1 (14.3)	3 (0.36–24.92)	
	Civil servant	11 (2.8)	10 (91)	1 (9)	1.26 (1.11–1.77)	
Religion	Orthodox^a^	6 (1.5)	4 (66.7)	2 (33.3)	1.00 (0.18–5.46)	0.13
	Catholic	1 (0.2)	1 (100)	0 (0)	---	
	Protestant	212 (53.3)	166 (78.3)	46 (21.7)	1.81 (1.3–2.5)	
	Muslims	178 (45)	124 (69.7)	54 (30.3)	1.15 (0.84–1.58)	
Receiving information related to malaria	No^a^	14 (3.5)	8 (57.1)	6 (42.9)	1.00 (0.35–2.89)	0.14
	Yes	384 (96.5)	287 (75)	96 (25)	2.25 (1.78–2.84)	

^a^reference category

### Knowledge about malaria and ITNs

**Fig. 2:**
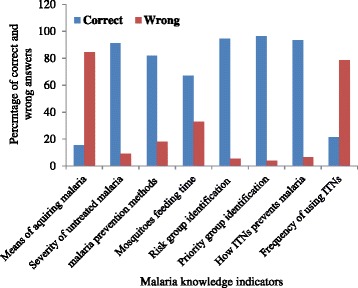
Percentage of knowledge about malaria and its prevention methods among pregnant mothers in Shashogo Woreda

Two hundred sixty one (65.6 %) women responded that malaria is transmitted due to poor personal hygiene and environmental sanitation whereas seventy-five (18.8 %) of them said malaria is acquired because of bad season. Only 62 (15.6 %) of them responded that malaria is transmitted through mosquito bite. The majority of the respondents 362 (91 %) was aware of the consequences of untreated malaria and 326 (81.9 %) of them mentioned ITNs as one of malaria prevention methods. Almost two third, 267 (67 %) of the pregnant women reported that malaria mosquitoes feed during night time and 376 (94.5 %) of them correctly identified high risk groups to malaria i.e., under five children or pregnant women to prioritize them for the disease prevention. Only eighty-five of the mothers (21.4 %) used ITNs every night rather majority of them 312 (78.6 %) used it seasonally particularly during cold season (Fig. 2).

The pregnant women’s overall level of knowledge about malaria and its prevention methods like ITNs was categorized as good or poor. Consequently, almost three fourth, 295 (74.3 %) of them had good knowledge while the remaining 25.7 % of the mothers possessed poor knowledge. Even though women of the age group 26–35 had a bit higher percentage of good knowledge (76.7 %) than the other two age groups i.e., 15–25 (71.7 %) and 36–45 (71 %), the association was not statistically significant (*P* = 0.85) (Table 1). Similarly, in terms of receiving information about malaria and ITNs, 74.9 % of the mothers who received information had good knowledge which is more than the percentage of good knowledge by those mothers who did not receive information (57.1 %) but the difference was not statistically significant (*P* = 0.14).

### Attitude towards malaria and ITNs

Slightly more than half of the pregnant women 203 (51.1 %) had positive attitude towards malaria and ITNs. Women of the younger age group i.e., 15–25 possessed significantly high percentage of positive attitude (60 %) as compared to other age groups, 26–35 (50.7 %) and 36–45 (35.5 %) in both univariate and multivariate analysis (*P* = 0.01) (Table 2). Likewise, mothers who obtained information about malaria and ITNs had significantly high frequency of positive attitude (52.2 %) than those who didn’t receive (21.4 %) (*P* = 0.02). The source of information was also significantly associated with attitude towards malaria and ITNs. Information obtained from TV/Radio and health extension workers being more important to change attitude than the information from friends/neighbours (*P* = 0.003). Twenty out of twenty nine pregnant mothers (68 %) who attended secondary education or above had positive attitude which is higher than those who are illiterate (49 %), but the association was not statistically significant (*P* = 0.17).

**Table 2:** Multivariate analysis of association between attitude towards malaria and ITNs and some socio-demographic characteristics among pregnant women, Shashogo Woreda, Southern Ethiopia, 2014

Variables	Label	Frequency (%)	Positive	Negative	Adjusted	*P*-value
			n (%)	n (%)	OR (95 % CI)	
Age	15–25^a^	120 (30.2)	72 (60)	48 (40)	1.00 (0.69–1.44)	0.01*
	26–35	215 (54)	109 (50.7)	106 (49.3)	0.68 (0.53–0.89)	
	36–45	62 (15.8)	22 (35.5)	40 (64.5)	0.37 (0.22–0.62)	
Receiving information related to malaria	No^a^	14 (3.5)	3 (21.3)	11 (78.7)	1.00 (0.27–3.63)	0.002*
	Yes	383 (96.5)	200 (52.2)	183 (47.8)	4.04 (3.30–4.97)	
Source of information	Neighbors/friends^a^	31 (8)	7 (22.6)	24 (77.4)	1.00 (0.45–2.34)	0.003*
	Health institutions/health extension workers	336 (87.8)	183 (54.5)	153 (45.5)	4.14 (3.31–5.10)	
	Radio	16 (4.2)	10 (62.5)	6 (37.5)	5.76 (2.10–15.83)	

^*^Statistically significant at *P* < 0.05

^a^reference category

### Practice towards ITNs

**Fig. 3:**
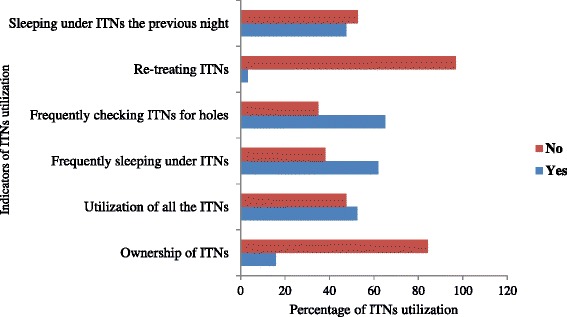
Utilization of ITNs by pregnant women in Shashogo Woreda, Southern Ethiopia, 2014

**Fig. 4:**
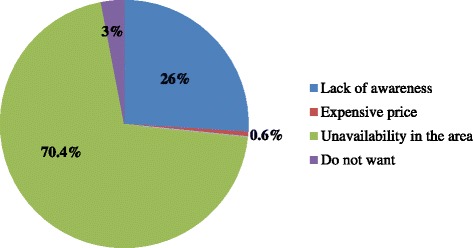
Reasons not to own ITNs by pregnant women in Shashogo Woreda, Southern Ethiopia, 2014

Only sixty-three (15.8 %) of the participants owned at least one ITNs (Fig. 3). The main reasons reported for this was unavailability in local markets (74.4 %) (Fig. 4). More than half of the pregnant women who owned ITNs 33 (52.4 %) had poor practice of ITNs utilization. Similarly, almost half 29 (46 %) of the mothers who possessed ITNs had six or more family members though maximum number of ITNs they owned was only two, and nearly half of them (47.6 %) reported that they were not using it all. A little more than half (52 %) of the mothers who had ITNs had not slept under bed net the previous night of the interview date. Almost all of the pregnant women (96.8 %) who owned ITNs had never re-treated their ITNs mainly because of the absence of K-O tab in the area.

Level education was significantly associated with ITNs utilization among the pregnant mothers with 86.7 % of mothers attending secondary education or above had good practice (*P* = 0.01) (Table 3). There was also considerable difference in ITNs utilization according to the respondents’ occupation. In this regard civil servants had absolutely good practice (100 %) but the difference was not statistically significant in multivariate analysis (*P* = 0.28). In the same way pregnant women who had good knowledge about malaria and ITNs also had relatively good practice of ITNs utilization (52.9 %) as compared to those who had poor knowledge (25 %) though the association was not statistically significant (*P* = 0.09) (Table 4).

**Table 3:** Multivariate analysis of association between practice of ITNs and some socio-demographic characteristics among pregnant women, Shashogo Woreda, Southern Ethiopia, 2014

Variables	Label	Frequency (%)	Good	Poor	Adjusted	*P*-value
			n (%)	n (%)	OR (95 % CI)	
Education status	Illiterate^a^	23 (36.5)	5 (21.7)	18 (78.3)	1.00 (0.36–2.68)	0.01^*^
	Primary (1–8)	25 (39.7)	12 (48)	13 (52)	3.29 (1.50–7.21)	
	Secondary and above	15 (23.8)	13 (86.7)	2 (13.3)	23.21 (5.25–102.86)	
Occupation	Student^a^	1 (1.6)	1 (100)	0 (0)	----	0.28
	Housewife	50 (79.4)	20 (40)	30 (60)	1.00 (0.57–1.75)	
	Self employed	5 (7.9)	2 (40)	3 (60)	1.00 (0.16–5.96)	
	Civil servant	7 (11.1)	7 (100)	0 (0)	----	

^*^Statistically significant at *P* < 0.05

^a^reference category

**Table 4:** Univariate analysis of association between ITNs utilization and knowledge and attitude towards malaria and ITNs among pregnant women, Shashogo Woreda, Southern Ethiopia, 2014

Variable	Level	Frequency (%)	Good (%)	Poor (%)	Crude OR (95 % CI)	P-value
Knowledge	Poor^a^	12 (19)	3 (25)	9 (75)	1.00 (0.27–3.73)	0.09
	Good	51 (81)	27 (53)	24 (47)	3.42 (1.97–5.91)	
Attitude	Negative^a^	19448.9	8 (57.1)	6 (42.9)	1.00 (0.35–2.89)	0.42
	Positive	20351.1	22 (44.9)	27 (55.1)	0.61 (0.35–1.10)	

^a^reference category

## Discussion

The current study revealed that pregnant women’s overall knowledge and attitude about malaria and ITNs in Shashogo Woreda was relatively good whereas their ITNs ownership and utilization was noticeably poor.

Except the means of transmission of malaria to which only 15.6 % of the mothers said it is through mosquitoes bite, most of the pregnant women provided the right response for the majority of malaria knowledge questions, such as severity of the disease, prevention methods, malaria mosquito feeding time, mechanisms of how ITNs prevents malaria, and high risk and priority group identification. Similarly, more than half of the pregnant women’s population showed positive attitude towards severity of malaria and its prevention methods like sleeping under bed nets as well as its threat on under five children and pregnant women.

The correct response about the means of malaria transmission in this study was remarkably lower than the values reported by other studies in Ethiopia and elsewhere in Africa [15–17], while the other outcomes mentioned above are similar with the findings of these studies. The possible explanation for the very poor knowledge about the transmission means could be misunderstanding attributed to the attention they gave for personal hygiene and environmental sanitation as they had been told by health extension workers and that was possibly why 65.6 % of them said it is due to poor personal hygiene and environmental sanitation. Nevertheless, poor knowledge of the community with regard to the link between mosquitoes and malaria in Ethiopia was already documented and it is found to be one of the major challenges in malaria prevention and control process in the country [18].

The 51.1 % positive attitude recorded in this study is more than the one reported from Nigeria (30 %) [19]. Mothers who got information from health extension workers and TV/radio had significantly better attitude than those who obtained information from neighbours or friends. This could be the result of more accurate information they obtained from health extension workers and radio. Pregnant women’s age was also found to be determining factor for their attitude in this study, younger age groups having better attitude than the others which is in contrary with studies in Northern Ethiopia and Nigeria [15, 19]. However, in another study in Nigeria age significantly affected women’s attitude towards ITNs [17].

As WHO and RBM recommend, regular and timely use of long-lasting, insecticide-treated nets (LLINs) is one of the main malaria prevention strategies in pregnancy [3, 20]. In contrast to this, ownership and utilization of ITNs by pregnant women in Shashogo Woreda was poor. Only 15.8 % owned ITNs which is by far lower than the 2011 national report which declared more than four million ITNs distribution to cover 90 % households with ≥1 ITNs and 63 % of the population at risk [9]. The main reasons reported for this was unavailability in local markets and unsustainable distribution. About one quarter of them reported that they had no information about ITNs as it is important method to prevent malaria. Similar findings were reported from different studies in sub-Saharan Africa where unavailability, inconsistency of distribution, cost, and failure to issue vouchers were frequently identified as barriers to ownership [21–23].

Moreover, to make the matter worst more than half of the mothers who owned the ITNs had no enough number proportional to their family size, did not use it all, had never checked it for holes, were using it occasionally, and had not slept under bed net the previous night of the interview date. The main reasons reported for this was due to its being dirty, old (served for more than years), had openings and in some cases lack of awareness on how to mount and its importance to prevent malaria. This huge miss use identified in this study confirmed the very low use of ITNs repeatedly reported from different African countries [24–27].

The present study also found that level of education was significant predictor of ITNs use, which is concordant with the findings of the studies in Northern Ethiopia and Kenya [28, 15]. The study in Kenya also showed that level of knowledge about ITNs was another determinant factor for its utilization [28] but it was not in this study despite the high percentage of good practice among mothers who had good knowledge. This might be due to very low number of study participants who owned ITNs, which might hinder the analysis of ITNs practice with respect to various factors which had been done for knowledge assessment.

## Conclusion

The current study identified that pregnant women’s knowledge and attitude about malaria and ITNs in this community was fairly good. However, they were poorly utilizing ITNs. This was similar with the finding of different studies [16, 19] and it shows that having awareness and knowledge merely doesn’t guarantee practice of intervention methods. The main causes for the low use of ITNs were low ownership due to inaccessibility and lack of regular use because of its exhaustion as well as lack of awareness about its importance in some of the cases.
